# Home for the American Medical Association

**Published:** 1902-02

**Authors:** 


### Home for the American Medical Association.

The *St. Paul Medical Journal,* in an editorial upon the future of the American Medical Association, urges the Association's need of a permanent and fitting home in an appropriate building in one of the large cities, preferably Chicago. A million dollars so invested would pay a good return in interest. The building should provide accommodation for the editorial and mechanical work of the *Journal,* and should contain a library, reading and lounging rooms, a pathological museum, a hall of fame for the heroes of medicine, etc. The remainder of the building should be constructed for renting. R.

